# Triglyceride-to-HDL Cholesterol Ratio is Associated with Hepatic Steatosis but not Fibrosis in Patients with Severe Obesity: A Cross-Sectional Study

**DOI:** 10.1007/s11695-026-08891-z

**Published:** 2026-08-19

**Authors:** Carolina de Oliveira Bernardes, Esdras Marques Lins, Fernanda Appolonio Rocha, Flavio Kreimer, Roberta Bessa Veloso Silva, Nataly Regina Fonseca Carvalho de Medeiros, Karolyna Maria Lima de Medeiros Ferreira, Alvaro Antonio Bandeira Ferraz

**Affiliations:** 1 Postgraduated Program in Surgery, Federal University of Pernambuco, Recife, Brazil; 2 Department of Surgery, Federal University of Pernambuco, Recife, Brazil; 3 Instituto de Ciências Exatas e Biológicas, Universidade José do Rosário Vellano, Alfenas, Brazil; 4 Agência Brasileira de Apoio a Gestão do SUS, São Paulo, Brazil; 5 Ministry of Health, São Paulo, Brazil

**Keywords:** Metabolic dysfunction-associated steatotic liver disease, Hepatic steatosis, Triglyceride-to-HDL cholesterol ratio, Insulin resistance, Severe obesity, Bariatric surgery

## Abstract

**Background:**

Metabolic dysfunction-associated steatotic liver disease (MASLD) is highly prevalent among individuals with severe obesity. The triglyceride-to-high-density lipoprotein cholesterol ratio (TG/HDL-C) is an accessible surrogate marker of insulin resistance, but its association with hepatic steatosis and fibrosis in candidates for metabolic and bariatric surgery remains incompletely defined.

**Methods:**

This retrospective cross-sectional study included 99 patients undergoing preoperative evaluation for metabolic and bariatric surgery between 2020 and 2024. Hepatic steatosis was assessed by ultrasonography and liver fibrosis by elastography. Associations with TG/HDL-C were examined using categorical analyses and multivariable logistic regression. Because diabetes perfectly predicted hepatic steatosis, Firth penalized logistic regression was used for the primary adjusted analysis.

**Results:**

The cohort included 61 women (61.6%), with a mean age of 37.0 ± 9.8 years and body mass index of 40.4 ± 4.2 kg/m². Hepatic steatosis was present in 81 of 99 patients (81.8%), whereas fibrosis was identified in 35 of 98 patients with evaluable elastography (35.7%). Higher TG/HDL-C categories were associated with hepatic steatosis (*p* = 0.0016). After adjustment, each unit increase in TG/HDL-C was associated with higher odds of steatosis (adjusted odds ratio, 2.23; 95% CI, 1.22–4.08; *p* = 0.0090). TG/HDL-C showed moderate discrimination for steatosis (area under the curve, 0.763; 95% confidence interval, 0.632–0.868), with an exploratory optimal threshold of 2.17 (79.0% sensitivity and 66.7% specificity). In contrast, TG/HDL-C was not associated with fibrosis after adjustment (adjusted odds ratio, 0.84; 95% confidence interval, 0.63–1.10; *p* = 0.209) and showed no discriminatory ability for fibrosis (area under the curve, 0.494).

**Conclusions:**

TG/HDL-C was independently associated with ultrasonographically detected hepatic steatosis and showed moderate discriminatory performance in patients with severe obesity undergoing evaluation for metabolic and bariatric surgery. However, it was not associated with fibrosis assessed by elastography. These findings support TG/HDL-C as a potential low-cost adjunct for identifying steatosis, but not as a substitute for fibrosis-specific assessment.

## Introduction

Metabolic dysfunction-associated steatotic liver disease (MASLD) is currently recognized as the most prevalent chronic liver disease worldwide, affecting approximately 25% to 30% of the global population, with even higher rates among individuals with obesity [1, 2]. In addition to its high prevalence, MASLD represents a major cause of liver-related morbidity and mortality and may progress to advanced fibrosis, cirrhosis, and hepatocellular carcinoma [3]. Patients with severe obesity represent a particularly susceptible population to MASLD.

Despite its clinical relevance, MASLD often follows a silent course and is asymptomatic in most cases. As a result, the disease is frequently identified incidentally through abnormalities in liver enzymes or imaging findings, reinforcing the need for more accessible and effective screening strategies [4].

Early identification of hepatic steatosis, particularly its progression to fibrosis, is essential to reduce adverse outcomes. Liver biopsy remains the gold standard for diagnosis [5]; however, it is invasive and has limited applicability in clinical practice. Imaging methods, such as computed tomography and magnetic resonance imaging, can also be used [4], but they have limitations related to cost and, in the case of computed tomography, exposure to ionizing radiation.

Abdominal ultrasonography has therefore become the most widely used non-invasive method in clinical practice due to its low cost and wide availability. In addition, liver elastography allows the assessment of hepatic tissue stiffness, estimating the degree of fibrosis based on the propagation velocity of ultrasound waves [6]. However, methodological variability and operator dependence may limit its accuracy, particularly in individuals with obesity [7, 8].

From a pathophysiological standpoint, MASLD is directly associated with hepatic triglyceride accumulation, resulting from alterations in lipid metabolism and insulin resistance [9]. In this context, simple laboratory markers such as the triglyceride-to-HDL ratio (TG/HDL) have been proposed as potential indicators of hepatometabolic risk [10]. Given that individuals with severe obesity are at higher risk for MASLD, investigating accessible biomarkers is particularly relevant in this population [11].

In this context, this study aimed to investigate the association of the triglyceride-to-HDL cholesterol ratio (TG/HDL-C) with hepatic steatosis assessed by ultrasonography and liver fibrosis assessed by elastography in patients with severe obesity undergoing preoperative evaluation for metabolic and bariatric surgery. We additionally evaluated whether TG/HDL-C was independently associated with these hepatic outcomes and explored its discriminatory performance and optimal threshold for detecting hepatic steatosis and fibrosis.

## Methods

### Study Design and Setting

This retrospective cross-sectional study was conducted using data obtained from the electronic medical records of patients evaluated for metabolic and bariatric surgery between January 2020 and December 2024 at XXXXXXXXXXXXXXXXXXXXXXX. All clinical, laboratory, ultrasonographic, and elastographic data were obtained during the preoperative assessment. The study was approved by the institutional Human Research Ethics Committee (approval no. 7,835,810; CAAE: 90542125.1.0000.8807). The requirement for individual informed consent was waived because of the retrospective study design and use of deidentified data.

### Study Population

Eligible participants were patients aged 18 years or older with a body mass index (BMI) ≥ 35 kg/m² who underwent standardized preoperative evaluation for metabolic and bariatric surgery during the study period. Inclusion required available anthropometric measurements, serum triglyceride and HDL-C concentrations, liver biochemistry, platelet count, glycated hemoglobin, abdominal ultrasonography, and liver shear-wave elastography.

Exclusion criteria included serologic evidence or a documented history of viral hepatitis, excessive alcohol consumption, incomplete data required for calculation of the TG/HDL-C ratio, absence of abdominal ultrasonography, and absence of a valid liver elastographic examination. Patients without a valid elastographic classification were excluded only from fibrosis-specific analyses. Individuals with incomplete or inconsistent data that precluded classification of the primary outcome were also excluded.

### Clinical and Laboratory Assessment

Demographic and anthropometric variables included age, sex, body weight, height, and BMI. BMI was calculated as weight in kilograms divided by height in meters squared.

Laboratory variables included total cholesterol, low-density lipoprotein cholesterol, very-low-density lipoprotein cholesterol, HDL-C, triglycerides, fasting glucose, glycated hemoglobin, insulin, aspartate aminotransferase (AST), alanine aminotransferase (ALT), albumin, and platelet count. Laboratory measurements were obtained during the preoperative evaluation according to routine institutional procedures.

The TG/HDL-C ratio was calculated by dividing fasting serum triglyceride concentration by HDL-C concentration, both expressed in mg/dL. TG/HDL-C was analyzed primarily as a continuous variable. For exploratory categorical analyses, values were classified as < 1.7, 1.7–3.0, and > 3.0. Because thresholds reported in the literature vary according to population, sex, ethnicity, and metabolic context, these categories were not interpreted as universal diagnostic cutoffs. A data-derived threshold was additionally estimated using receiver operating characteristic analysis and the Youden index.

### FIB-4 Calculation

The Fibrosis-4 index (FIB-4) was calculated using the following formula:

$$\begin{array}{c}\mathrm{FIB}-4=\left[\mathrm{Age}\left(\mathrm{years}\right)\times\mathrm{AST}\left(\mathrm U/\mathrm L\right)\right]/\\\left[\mathrm{Platelet}\left(10^9/\mathrm L\right)\times\sqrt{}\mathrm{ALT}\left(\mathrm U/\mathrm L\right)\right]\end{array}$$

FIB-4 values < 1.3 were classified as low risk, whereas values ≥ 1.3 were classified as elevated. Participants with missing age, AST, ALT, or platelet count were excluded from FIB-4–specific analyses. FIB-4 was considered a complementary fibrosis risk-stratification tool and not a diagnostic reference standard.

### Ultrasonographic Assessment of Hepatic Steatosis

Hepatic steatosis was assessed by conventional B-mode abdominal ultrasonography performed using an Aplio system (Canon Medical Systems, Otawara, Japan) or a LOGIQ system (GE HealthCare, Chicago, IL, USA). Steatosis was classified as absent, mild, moderate, or severe based on liver echogenicity relative to the renal cortex, visualization of the intrahepatic vessels and diaphragm, and posterior beam attenuation. For the primary analysis, hepatic steatosis was defined as the presence of mild, moderate, or severe steatosis. Moderate and severe steatosis were combined for a secondary analysis.

### Liver Elastography

Liver stiffness was assessed using point shear-wave elastography or two-dimensional shear-wave elastography and was expressed in kilopascals (kPa). A minimum of six valid measurements was required for each examination. Measurement reliability was assessed using the ratio of the interquartile range to the median liver stiffness value (IQR/median), with values < 30% considered indicative of an acceptable-quality examination. Liver fibrosis was categorized as F0 (no fibrosis), F1 (portal fibrosis), F2 (fibrosis with few septa), F3 (advanced fibrosis), or F4 (cirrhosis), according to the liver stiffness thresholds adopted by the imaging service. For the analyses, any fibrosis was defined as F1–F4 and significant fibrosis as F2–F4. Patients without an examination meeting the predefined quality criteria were excluded from fibrosis-specific analyses.

### Study Outcomes

The primary outcome was the presence of hepatic steatosis on ultrasonography. Secondary outcomes were moderate-to-severe steatosis, any fibrosis (F1–F4), and significant fibrosis (F2–F4) assessed by liver elastography. The principal exposure was the TG/HDL-C ratio, analyzed continuously and in predefined exploratory categories.

### Sample Size

The sample size was estimated for a cross-sectional prevalence analysis using a conservative expected prevalence of 50%, a 95% confidence level, and an absolute precision of 10%. After finite-population correction for approximately 1,600 potentially eligible patients during the study period, the minimum estimated sample was 91 participants. The final sample of 99 patients exceeded this requirement.

This calculation was intended to support prevalence estimation and was not specifically powered to detect small effect sizes in multivariable fibrosis analyses.

### Statistical Analysis

Continuous variables were summarized as mean and standard deviation or median and interquartile range, according to their distribution. Categorical variables were reported as absolute frequencies and percentages. Percentages were presented to one decimal place. No inferential p values were calculated for single-sample descriptive distributions.

Associations between TG/HDL-C categories and hepatic outcomes were evaluated using the chi-square test or Fisher’s exact test, as appropriate. Exact p values were reported.

Multivariable logistic regression was used to evaluate whether TG/HDL-C, analyzed as a continuous variable, was independently associated with hepatic steatosis and fibrosis. The prespecified adjustment model included age, sex, BMI, and diabetes mellitus. Because all patients with diabetes had hepatic steatosis, resulting in complete separation, Firth penalized logistic regression was used for the primary steatosis model. An expanded sensitivity model additionally included glycated hemoglobin. Conventional logistic regression was retained for outcomes without separation. Results were reported as odds ratios with 95% confidence intervals.

Receiver operating characteristic curves were constructed to assess the ability of the TG/HDL-C ratio to discriminate between patients with and without each hepatic outcome. AUCs were estimated with bootstrap 95% confidence intervals based on 1,000 resamples. For outcomes showing discriminatory ability, exploratory optimal thresholds were identified by maximizing the Youden index, and the corresponding sensitivity and specificity were calculated. Because threshold selection and performance estimation were conducted in the same sample, these results were considered exploratory and potentially optimistic and require external validation.

Analyses of fibrosis were conducted using complete cases with valid elastographic classifications. Missing values were not imputed. Principal component analysis was removed because it did not directly address the prespecified clinical objectives. All tests were two-sided, and *p* < 0.05 was considered statistically significant. All statistical analyses were performed using IBM SPSS v18. [12].

## Results

### Study Population

A total of 99 patients undergoing preoperative evaluation for metabolic and bariatric surgery were included. The cohort comprised 61 women (61.6%) and 38 men (38.4%). Age was available for 97 participants, with a mean of 37.0 ± 9.8 years. Mean BMI was 40.4 ± 4.2 kg/m². The median TG/HDL-C ratio was 2.93 (interquartile range [IQR], 1.99–4.01). Fifteen patients (15.2%) had a TG/HDL-C ratio < 1.7, 37 (37.4%) had values between 1.7 and 3.0, and 47 (47.5%) had values > 3.0 (Table 1).

**Table 1:** Characteristics of the study population

Variable	Value
Participants	99
Women	61 (61.6%)
Men	38 (38.4%)
Age, years	37.0 ± 9.8
BMI, kg/m²	40.4 ± 4.2
Total cholesterol, mg/dL	193 (171.5–213.5)
LDL-C, mg/dL	123 (106–137.4)
HDL-C, mg/dL	44 (40–49.5)
Triglycerides, mg/dL	134 (95.5–167)
TG/HDL-C ratio	2.93 (1.99–4.01)
AST, U/L	26 (19–36.8)
ALT, U/L	35 (20–57)
Platelet count, cells ×10⁹/L	285 (246–320.5)
FIB-4	0.57 (0.40–0.79)
Glycated hemoglobin, %	5.6 (5.4–6.0)
Fasting glucose, mg/dL	96 (89–104.5)
Insulin	26.5 (18.9–37.5)
TG/HDL-C < 1.7	15 (15.2%)
TG/HDL-C 1.7–3.0	37 (37.4%)
TG/HDL-C > 3.0	47 (47.5%)
Any hepatic steatosis	81 (81.8%)
Moderate-to-severe steatosis	71 (71.7%)
Any fibrosis, F1–F4	35/98 (35.7%)
Significant fibrosis, F2–F4	30/98 (30.6%)
FIB-4 ≥ 1.3	6/95 (6.3%)

Data are presented as mean ± standard deviation, median (interquartile range), or n (%). Denominators are provided when data were unavailable for the full cohort. BMI, body mass index; AST, aspartate aminotransferase; ALT, alanine aminotransferase

### Hepatic Steatosis and TG/HDL-C

Hepatic steatosis was identified by ultrasonography in 81 of 99 participants (81.8%). Steatosis was mild in 10 patients (10.1%), moderate in 39 (39.4%), and severe in 32 (32.3%). The remaining 18 patients (18.2%) had no ultrasonographic evidence of steatosis.

The prevalence of hepatic steatosis increased progressively across TG/HDL-C categories, from 53.3% among patients with TG/HDL-C < 1.7 to 78.4% among those with values between 1.7 and 3.0 and 93.6% among those with TG/HDL-C > 3.0 (*p* = 0.0016). Moderate-to-severe steatosis was present in 40.0%, 73.0%, and 80.9% of these groups, respectively (*p* = 0.0091) (Table 2).

**Table 2:** Hepatic outcomes according to TG/HDL-C category

Outcome	< 1.7 (*n* = 15)	1.7–3.0 (*n* = 37)	> 3.0 (*n* = 47)	*p* value
No steatosis	7 (46.7%)	8 (21.6%)	3 (6.4%)	
Mild steatosis	2 (13.3%)	2 (5.4%)	6 (12.8%)	
Moderate steatosis	4 (26.7%)	18 (48.6%)	17 (36.2%)	
Severe steatosis	2 (13.3%)	9 (24.3%)	21 (44.7%)	
Any steatosis	8 (53.3%)	29 (78.4%)	44 (93.6%)	0.0016
Moderate-to-severe steatosis	6 (40.0%)	27 (73.0%)	38 (80.9%)	0.0091
Any fibrosis, F1–F4	4/15 (26.7%)	14/37 (37.8%)	17/46 (37.0%)	0.727
Significant fibrosis, F2–F4	4/15 (26.7%)	13/37 (35.1%)	13/46 (28.3%)	0.746

Percentages are calculated within TG/HDL-C categories. One patient in the > 3.0 category did not have a valid elastographic classification. The p values for any and moderate-to-severe steatosis refer to comparisons across the three TG/HDL-C categories

When TG/HDL-C was dichotomized at 1.7, values > 1.7 were associated with higher odds of any hepatic steatosis (OR, 5.81; *p* = 0.0055) and moderate-to-severe steatosis (OR, 5.13; *p* = 0.0097).

### Multivariable Analysis of Hepatic Steatosis

In unadjusted logistic regression, each unit increase in TG/HDL-C was associated with higher odds of hepatic steatosis (OR, 2.31; 95% CI, 1.34–3.97; *p* = 0.0025). This association remained significant after adjustment for age, sex, and BMI (adjusted OR [aOR], 2.75; 95% CI, 1.44–5.25; *p* = 0.0022) and after additional adjustment for glycated hemoglobin (aOR, 2.73; 95% CI, 1.36–5.49; *p* = 0.0047) (Table 3).

**Table 3:** Association of TG/HDL-C with hepatic outcomes

Outcome and model	OR per 1-unit increase	95% CI	*p* value
Any hepatic steatosis			
Unadjusted	2.31	1.34–3.97	0.0025
Adjusted for age, sex, and BMI	2.75	1.44–5.25	0.0022
Adjusted for age, sex, BMI, and HbA1c	2.73	1.36–5.49	0.0047
Firth model adjusted for age, sex, BMI and diabetes	2.23	1.22–4.08	0.0090
Expanded Firth model additionally adjusted for HbA1c	2.24	1.21–4.14	0.0099
Moderate-to-severe steatosis			
Unadjusted	1.46	1.04–2.05	0.027
Adjusted for age, sex, and BMI	1.55	1.05–2.27	0.026
Adjusted for age, sex, BMI, and HbA1c	1.37	0.91–2.05	0.127
Any fibrosis, F1–F4			
Unadjusted	0.92	0.74–1.14	0.444
Adjusted for age, sex, and BMI	0.92	0.72–1.18	0.501
Adjusted for age, sex, BMI, and HbA1c	0.84	0.63–1.10	0.209
Significant fibrosis, F2–F4			
Unadjusted	0.89	0.70–1.13	0.332
Adjusted for age, sex, and BMI	0.88	0.67–1.16	0.381
Adjusted for age, sex, BMI, and HbA1c	0.83	0.61–1.12	0.223

ORs refer to each one-unit increase in TG/HDL-C. HbA1c, glycated hemoglobin

TG/HDL-C was also associated with moderate-to-severe steatosis in the unadjusted analysis (OR, 1.46; 95% CI, 1.04–2.05; *p* = 0.027) and after adjustment for age, sex, and BMI (aOR, 1.55; 95% CI, 1.05–2.27; *p* = 0.026). However, this association was attenuated after glycated hemoglobin was added to the model (aOR, 1.37; 95% CI, 0.91–2.05; *p* = 0.127).

All 20 patients with diabetes had hepatic steatosis, resulting in complete separation in conventional logistic regression. Therefore, Firth penalized logistic regression was used for the primary adjusted analysis. After adjustment for age, sex, BMI, and diabetes, each unit increase in TG/HDL-C remained associated with higher odds of hepatic steatosis (aOR, 2.23; 95% CI, 1.22–4.08; *p* = 0.0090). The association remained significant in the expanded model additionally including glycated hemoglobin (aOR, 2.24; 95% CI, 1.21–4.14; *p* = 0.0099).

### Liver Fibrosis and TG/HDL-C

Liver elastography was evaluable in 98 patients. Fibrosis was absent (F0) in 63 patients (64.3%), whereas 5 (5.1%) had F1, 12 (12.2%) had F2, 9 (9.2%) had F3, and 9 (9.2%) had F4 fibrosis. Overall, any fibrosis (F1–F4) was present in 35 patients (35.7%), and significant fibrosis (F2–F4) was present in 30 (30.6%).

The prevalence of any fibrosis did not differ across TG/HDL-C categories: 26.7% in patients with TG/HDL-C < 1.7, 37.8% in those with values between 1.7 and 3.0, and 37.0% in those with values > 3.0 (*p* = 0.727). Similarly, no association was observed with significant fibrosis (*p* = 0.746) (Table 2).

TG/HDL-C was not associated with any fibrosis in the unadjusted model (OR, 0.92; 95% CI, 0.74–1.14; *p* = 0.444), after adjustment for age, sex, and BMI (aOR, 0.92; 95% CI, 0.72–1.18; *p* = 0.501), or after additional adjustment for glycated hemoglobin (aOR, 0.84; 95% CI, 0.63–1.10; *p* = 0.209). No association was observed between TG/HDL-C and significant fibrosis in the fully adjusted model (aOR, 0.83; 95% CI, 0.61–1.12; *p* = 0.223) (Table 3).

### Discriminatory Performance of TG/HDL-C

**Fig. 1:**
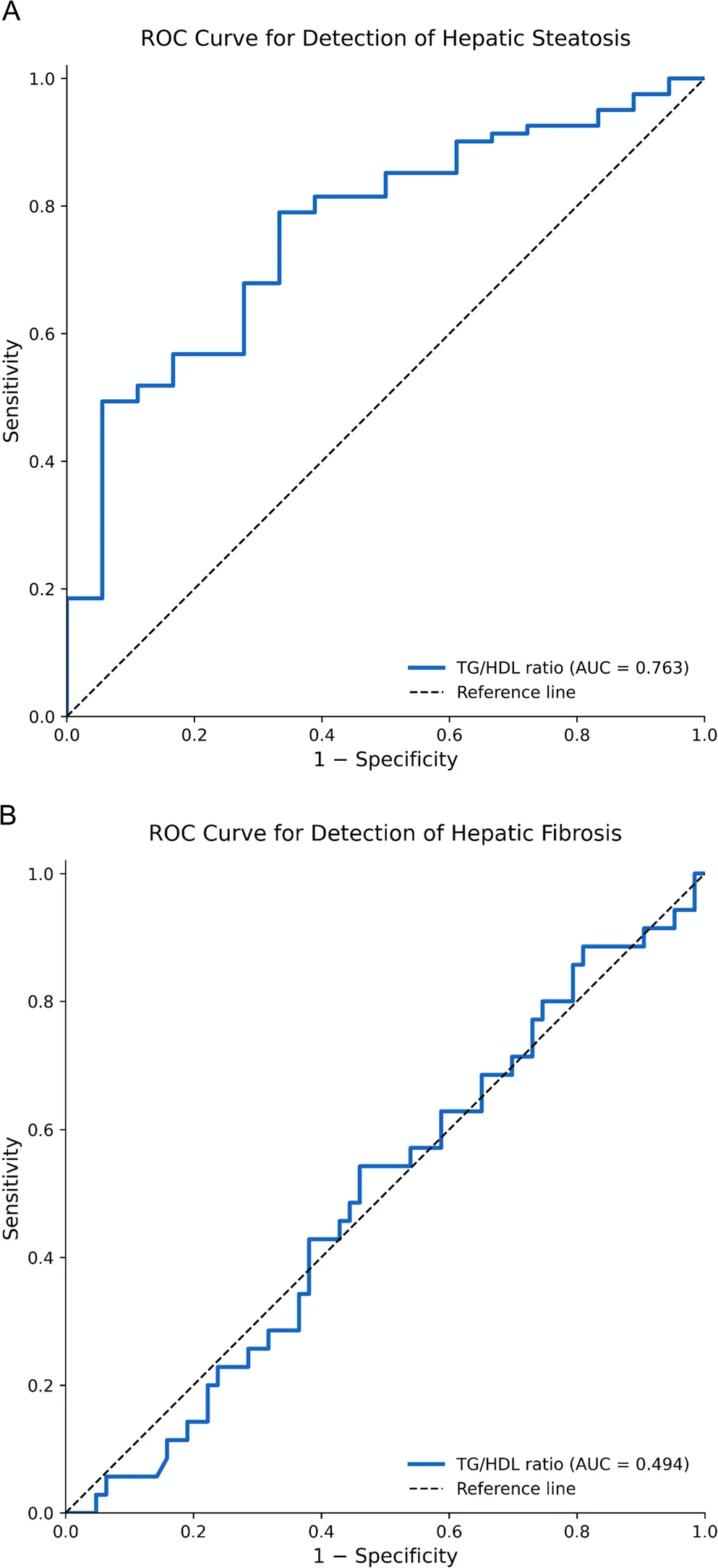
Receiver operating characteristic curves for the TG/HDL-C ratio. (A) Discrimination of any hepatic steatosis on ultrasonography (*n* = 99; AUC, 0.763; bootstrap 95% CI, 0.632–0.868). The exploratory threshold maximizing the Youden index was 2.17, with 79.0% sensitivity and 66.7% specificity. (B) Discrimination of any elastographic fibrosis (F1–F4 versus F0; *n* = 98; AUC, 0.494; bootstrap 95% CI, 0.379–0.612). AUC indicates area under the receiver operating characteristic curve; CI, confidence interval; TG/HDL-C, triglyceride-to–high-density lipoprotein cholesterol ratio

The TG/HDL-C ratio showed moderate discrimination for any hepatic steatosis, with an AUC of 0.763 (bootstrap 95% CI, 0.632–0.868; Fig. 1A). The exploratory threshold maximizing the Youden index was 2.17, corresponding to 79.0% sensitivity and 66.7% specificity. Discrimination was lower for moderate-to-severe steatosis (AUC, 0.668; 95% CI, 0.548–0.792). In contrast, TG/HDL-C did not discriminate patients with any fibrosis from those without fibrosis (AUC, 0.494; 95% CI, 0.379–0.612; Fig. 1B) and showed similarly poor discrimination for significant fibrosis (AUC, 0.454; 95% CI, 0.335–0.583) (Table 4).

**Table 4:** Discriminatory performance of TG/HDL-C

Outcome	AUC	Bootstrap 95% CI	Optimal threshold	Sensitivity	Specificity
Any hepatic steatosis	0.763	0.632–0.868	2.17	79.0%	66.7%
Moderate-to-severe steatosis	0.668	0.548–0.792	2.17	80.3%	53.6%
Any fibrosis, F1–F4	0.494	0.379–0.612	—	—	—
Significant fibrosis, F2–F4	0.454	0.335–0.583	—	—	—

### FIB-4 and Elastographic Fibrosis

FIB-4 and elastography were jointly evaluable in 95 patients. FIB-4 was < 1.3 in 89 patients (93.7%) and ≥ 1.3 in 6 (6.3%). Among patients with elastographic fibrosis, 3 of 33 (9.1%) had FIB-4 ≥ 1.3, compared with 3 of 62 patients (4.8%) without fibrosis. This difference was not statistically significant (*p* = 0.415).

## Discussion

In this cross-sectional study of patients undergoing preoperative evaluation for metabolic and bariatric surgery, the TG/HDL-C ratio was independently associated with ultrasonographically detected hepatic steatosis and showed moderate discriminatory performance. The prevalence of steatosis increased progressively across TG/HDL-C categories, reaching 93.6% among patients with values > 3.0. This association remained significant in Firth penalized logistic regression after adjustment for age, sex, BMI, and diabetes. In contrast, TG/HDL-C was not associated with either any fibrosis or significant fibrosis assessed by shear-wave elastography and showed no discriminatory ability for these outcomes. These findings suggest that TG/HDL-C reflects the metabolic disturbances associated with hepatic fat accumulation but should not be interpreted as a fibrosis-specific marker.

The biological plausibility of the association with steatosis is supported by the central role of insulin resistance in MASLD [9, 10, 13]. Impaired insulin action increases adipose tissue lipolysis and free fatty acid delivery to the liver, promotes hepatic de novo lipogenesis, and alters very-low-density lipoprotein production and clearance [9, 10, 13]. These changes favor hypertriglyceridemia and lower HDL-C concentrations, resulting in an elevated TG/HDL-C ratio. Consequently, TG/HDL-C may represent an accessible integrated marker of the dyslipidemic phenotype accompanying insulin resistance [18]. However, it does not directly quantify insulin sensitivity. HOMA-IR incorporates fasting insulin and glucose [14], whereas the triglyceride-glucose index, TyG-BMI, and the metabolic score for insulin resistance (METS-IR) combine different metabolic and anthropometric components [19, 20]. TG/HDL-C has the practical advantage of requiring only two routinely available lipid measurements, but its thresholds vary across sex, ethnicity, and metabolic context and should not be considered interchangeable with these more specific insulin-resistance indices [18–20].

Our findings regarding steatosis are consistent with previous studies. Martínez-Montoro et al. evaluated patients with obesity using liver biopsy and found that higher TG/HDL-C was associated with MASLD, supporting its potential as a readily available metabolic indicator in this population [21]. Wang et al., in an analysis of 6,495 participants from the National Health and Nutrition Examination Survey, reported a progressive association between TG/HDL-C and steatotic liver disease after adjustment for demographic and metabolic covariates [22]. In that study, participants in the highest TG/HDL-C quartile had substantially higher odds of steatotic liver disease than those in the lowest quartile. Other studies have similarly reported associations between higher TG/HDL-C values, hepatic steatosis, insulin resistance, and adverse glycometabolic profiles [15, 23]. Our adjusted findings extend this evidence to a population specifically undergoing evaluation for metabolic and bariatric surgery.

The exploratory TG/HDL-C threshold of 2.17 showed 79.0% sensitivity and 66.7% specificity for the presence of steatosis. This threshold should be interpreted cautiously because it was derived and evaluated in the same relatively small sample and was not externally validated. It should therefore not replace imaging or be proposed as a definitive diagnostic cutoff. Its potential clinical value lies in identifying patients who may warrant closer hepatometabolic assessment when imaging is not readily available. Conversely, the lower AUC for moderate-to-severe steatosis and attenuation of the adjusted association after inclusion of glycated hemoglobin indicate that TG/HDL-C partly reflects the broader glycometabolic disturbance rather than the anatomical severity of liver disease alone [21–23].

The absence of an association between TG/HDL-C and fibrosis is an important finding. Fibrosis reflects cumulative hepatocellular injury, inflammation, stellate-cell activation, and extracellular matrix deposition and therefore cannot be expected to parallel a single contemporaneous lipid ratio [9, 10]. Our results are consistent with the NHANES study by Wang et al., in which the association between TG/HDL-C and fibrosis was no longer statistically significant after comprehensive adjustment for metabolic covariates [22]. Thus, TG/HDL-C may identify the metabolic environment associated with steatosis without providing information about whether structural liver injury and fibrosis have developed. This distinction explains why the ratio showed moderate discrimination for steatosis but an AUC close to 0.50 for fibrosis.

Contemporary fibrosis assessment increasingly relies on sequential and multimodal noninvasive strategies rather than a single serum marker [5, 8]. FIB-4 remains useful as an initial rule-out test, but its age-dependent formulation may produce low values among younger adults, including candidates for metabolic and bariatric surgery [16, 17]. In the present cohort, only 6.3% of patients had FIB-4 ≥ 1.3, although shear-wave elastography identified fibrosis in 35.7%. This discordance should not be interpreted as proof that elastography is more sensitive, because histologic confirmation was unavailable and early elastographic stages may be affected by technical and biological variability [7, 8]. Instead, it illustrates that FIB-4 and liver stiffness measurements evaluate different dimensions of fibrosis risk and that neither TG/HDL-C nor FIB-4 should be used in isolation.

Newer noninvasive tools provide a more fibrosis-oriented approach. The Fibrotic NASH Index combines AST, HDL-C, and glycated hemoglobin and was specifically developed to identify fibrotic steatohepatitis [24]. The FAST score integrates liver stiffness, controlled attenuation parameter, and AST [25]; the MAST score combines magnetic resonance imaging-derived proton-density fat fraction, magnetic resonance elastography, and AST [26]; and the Agile 3 + and Agile 4 scores incorporate liver stiffness with clinical and laboratory variables to estimate advanced fibrosis and cirrhosis [27]. These instruments serve a different purpose from TG/HDL-C. Whereas TG/HDL-C may function as an early, inexpensive marker of metabolic dysfunction and hepatic fat accumulation, fibrosis-oriented scores and elastography are required when the clinical question is structural liver injury. The Fibrotic NASH Index achieved an AUROC of 0.78 in its derivation cohort and AUROCs ranging from 0.80 to 0.95 in external validation cohorts [24].

These findings are particularly relevant to metabolic and bariatric surgery. In the preoperative setting, TG/HDL-C may complement routine metabolic assessment and help identify patients with a high likelihood of steatosis who may benefit from targeted imaging and optimization of cardiometabolic risk factors. However, it cannot determine the presence or stage of fibrosis, and patients with clinical suspicion of advanced liver disease should undergo fibrosis-specific assessment regardless of TG/HDL-C [5, 8]. Metabolic and bariatric surgery frequently leads to substantial improvement in steatosis, MASH, and fibrosis. In a prospective study with sequential liver biopsies, Lassailly et al. reported MASH resolution without worsening fibrosis in 84% of patients five years after surgery, with progressive fibrosis regression over time [28].

The potential postoperative role of TG/HDL-C is hypothesis-generating. Because it is inexpensive and routinely available, serial TG/HDL-C measurement could help characterize persistent or recurrent metabolic dysfunction after surgery, particularly in patients with insufficient weight loss, weight recurrence, recurrent diabetes, or dyslipidemia [29]. Such patients may also warrant renewed hepatic imaging, fibrosis assessment, nutritional and behavioral intervention, or adjunctive pharmacotherapy. Nevertheless, the present study included only preoperative cross-sectional data and did not evaluate postoperative trajectories, weight recurrence, MASH recurrence, or fibrosis progression. Therefore, longitudinal monitoring applications cannot be inferred from our results and should be investigated in future studies.

This study has several strengths. It evaluated a clinically relevant population at high risk for MASLD, included both ultrasonographic and shear-wave elastographic assessment, analyzed TG/HDL-C continuously and categorically, and incorporated multivariable and ROC analyses. The simultaneous evaluation of steatosis and fibrosis also allowed us to distinguish the metabolic information provided by TG/HDL-C from fibrosis-specific assessment.

Several limitations should be acknowledged. First, the retrospective cross-sectional design precludes conclusions regarding causality, disease progression, or prognostic performance. Second, this was a relatively small, single-center cohort selected from patients undergoing evaluation for metabolic and bariatric surgery, limiting generalizability to other populations. Third, liver biopsy was unavailable; therefore, ultrasonography and shear-wave elastography, rather than histology, were used to classify steatosis and fibrosis. Ultrasonography has limited sensitivity for mild steatosis and is operator dependent, while liver stiffness may be influenced by inflammation, congestion, technical factors, and severe obesity [6–8]. Fourth, elastography was performed using equipment from two manufacturers, introducing potential measurement heterogeneity despite the application of predefined quality criteria. Fifth, information regarding lipid-lowering medication use was not consistently available and may have influenced triglyceride and HDL-C concentrations. Sixth, the limited number of patients without steatosis and with elevated FIB-4 values resulted in imprecise estimates and restricted the complexity of multivariable models. Finally, the ROC-derived threshold was internally derived without external validation and should be regarded as exploratory.

Overall, TG/HDL-C may serve as a simple adjunctive indicator of hepatometabolic dysfunction and hepatic steatosis in patients undergoing evaluation for metabolic and bariatric surgery. Its accessibility could support the selection of patients for further liver assessment, particularly in resource-limited settings. However, its lack of association with fibrosis reinforces the need for fibrosis-specific algorithms, elastography, and, when clinically indicated, histologic evaluation [5, 8, 24–27].

## Data Availability

No datasets were generated or analysed during the current study.

## References

[1] Li Y, Yang P, Ye J, Xu Q, Wu J, Wang Y. Updated mechanisms of MASLD pathogenesis. Lipids Health Dis. 2024;23(1):117. 10.1186/s12944-024-02108-x. PMC11034170 38649999

[2] Younossi ZM, Koenig AB, Abdelatif D, Fazel Y, Henry L, Wymer M. Global epidemiology of nonalcoholic fatty liver disease—meta-analytic assessment of prevalence, incidence, and outcomes. Hepatology. 2016;64(1):73–84. 10.1002/hep.28431. 26707365

[3] Owrangi S, Paik JM, Golabi P, de Avila L, Hashida R, Nader A, et al. Meta-analysis: global prevalence and mortality of cirrhosis in metabolic dysfunction-associated steatotic liver disease. Aliment Pharmacol Ther. 2025;61(3):433–43. 10.1111/apt.18451. 39697043

[4] Chalasani N, Younossi Z, Lavine JE, Charlton M, Cusi K, Rinella M, et al. The diagnosis and management of nonalcoholic fatty liver disease: practice guidance from the American Association for the Study of Liver Diseases. Hepatology. 2018;67(1):328–57. 10.1002/hep.29367. 28714183

[5] Rinella ME, Neuschwander-Tetri BA, Siddiqui MS, Abdelmalek MF, Caldwell S, Barb D, et al. AASLD practice guidance on the clinical assessment and management of nonalcoholic fatty liver disease. Hepatology. 2023;77(5):1797–1835. 10.1097/HEP.0000000000000323 PMC10735173 36727674

[6] Hernaez R, Lazo M, Bonekamp S, Kamel I, Brancati FL, Guallar E, et al. Diagnostic accuracy and reliability of ultrasonography for the detection of fatty liver: a meta-analysis. Hepatology. 2011;54(3):1082–90. 10.1002/hep.24452. PMC4197002 21618575

[7] Eddowes PJ, Sasso M, Allison M, Tsochatzis E, Anstee QM, Sheridan D, et al. Accuracy of FibroScan controlled attenuation parameter and liver stiffness measurement in assessing steatosis and fibrosis in patients with nonalcoholic fatty liver disease. Gastroenterology. 2019;156(6):1717–30. 10.1053/j.gastro.2019.01.042. 30689971

[8] European Association for the Study of the Liver. EASL clinical practice guidelines on non-invasive tests for evaluation of liver disease severity and prognosis. J Hepatol 2021;75(3):659–689. 10.1016/j.jhep.2021.05.025 34166721

[9] Targher G, Valenti L, Byrne CD. Metabolic dysfunction–associated steatotic liver disease. N Engl J Med. 2025;393(7):683–98. 10.1056/NEJMra2412865. 40802944

[10] Friedman SL, Neuschwander-Tetri BA, Rinella M, Sanyal AJ. Mechanisms of NAFLD development and therapeutic strategies. Nat Med. 2018;24(7):908–22. 10.1038/s41591-018-0104-9. PMC6553468 29967350

[11] Navarro-Masip È, Mestres Petit N, Salinas-Roca B, Herrerías F, Vilardell F, de la Fuente MC, et al. Metabolic dysfunction-associated steatotic liver disease in severe obesity and concordance between invasive (biopsy) and noninvasive (OWLiver®) diagnoses. Obes Facts. 2024;17(5):473–82. 10.1159/000538765. PMC11540414 38934179

[12] R Core Team. R: A language and environment for statistical computing. Vienna, Austria: R Foundation for Statistical Computing; 2025. Available from: https://www.R-project.org/

[13] Grander C, Grabherr F, Tilg H. Non-alcoholic fatty liver disease: pathophysiological concepts and treatment options. *Cardiovasc Res*. 2023;119(9):1787–1798. 10.1093/cvr/cvad095. PMC10405569 37364164

[14] Matthews DR, Hosker JP, Rudenski AS, Naylor BA, Treacher DF, Turner RC. Homeostasis model assessment: insulin resistance and β-cell function from fasting plasma glucose and insulin concentrations in man. Diabetologia. 1985;28(7):412–419. 10.1007/BF00280883. 3899825

[15] Hoca E, Cangir B, Ahbab S, Şahin SI, Öztürk EÇ, Urvasızoğlu AÖ, et al. The triglyceride/HDL ratio as a non-invasive marker for early-stage NAFLD: a retrospective cross-sectional study of 2588 patients. Diagnostics (Basel). 2025;15(16):2045. 10.3390/diagnostics15162045. PMC12385797 40870897

[16] van Kleef LA, Sonneveld MJ, de Man RA, de Knegt RJ. Poor performance of FIB-4 in elderly individuals at risk for chronic liver disease - implications for the clinical utility of the EASL NIT guideline. J Hepatol. 2022;76(1):245–6. 10.1016/j.jhep.2021.08.017. 34461208

[17] Schwenger KJP, Alali M, Ghorbani Y, Fischer SE, Jackson TD, Okrainec A, et al. Reliability of non-invasive liver fibrosis assessment tools versus biopsy in pre- and post-bariatric surgery patients with non-alcoholic fatty liver disease. Obes Surg. 2023;33(1):247–55. 10.1007/s11695-022-06380-7. 36464738

[18] Gasevic D, Frohlich J, Mancini GBJ, Lear SA. The association between triglyceride to high-density-lipoprotein cholesterol ratio and insulin resistance in a multiethnic primary prevention cohort. Metabolism. 2012;61(4):583–589. 10.1016/j.metabol.2011.09.009. 22075272

[19] Bello-Chavolla OY, Almeda-Valdes P, Gomez-Velasco D, Viveros-Ruiz T, Cruz-Bautista I, Romo-Romo A, et al. METS-IR, a novel score to evaluate insulin sensitivity, is predictive of visceral adiposity and incident type 2 diabetes. Eur J Endocrinol. 2018;178(5):533–44. 10.1530/EJE-17-0883. 29535168

[20] Wang J, Yan S, Cui Y, Chen F, Piao M, Cui W. The diagnostic and prognostic value of the triglyceride-glucose index in metabolic dysfunction-associated fatty liver disease: a systematic review and meta-analysis. Nutrients. 2022;14(23):4969. 10.3390/nu14234969. PMC9741077 36500999

[21] Martínez-Montoro JI, Cornejo-Pareja I, Tinahones FJ, et al. Triglyceride to HDL cholesterol ratio for the identification of MASLD in obesity: a liver biopsy-based case-control study. Nutrients. 2024;16(9):1310. 10.3390/nu16091310. PMC11085202 38732557

[22] Wang J, Li H, Wang X, Shi R, Hu J, Zeng X, et al. Association between triglyceride to high-density lipoprotein cholesterol ratio and nonalcoholic fatty liver disease and liver fibrosis in American adults: an observational study from the National Health and Nutrition Examination Survey 2017–2020. Front Endocrinol (Lausanne). 2024;15:1362396. 10.3389/fendo.2024.1362396. PMC11286417 39081791

[23] Bian L, Tang T, Yu Q, et al. Association between the triglyceride to high-density lipoprotein cholesterol ratio and type 2 diabetes mellitus in non-alcoholic fatty liver disease. Sci Rep. 2024;14:31048. 10.1038/s41598-024-82116-0. PMC11680680 39730877

[24] Tavaglione F, Jamialahmadi O, De Vincentis A, et al. Development and validation of a score for fibrotic nonalcoholic steatohepatitis. Clin Gastroenterol Hepatol. 2023;21(6):1523-1532.e1. 10.1016/j.cgh.2022.03.044. 35421583

[25] Newsome PN, Sasso M, Deeks JJ, et al. FibroScan-AST (FAST) score for the non-invasive identification of patients with non-alcoholic steatohepatitis with significant activity and fibrosis: a prospective derivation and global validation study. Lancet Gastroenterol Hepatol. 2020;5(4):362–373. 10.1016/S2468-1253(19)30383-8. PMC7066580 32027858

[26] Noureddin M, Truong E, Gornbein JA, et al. MRI-based (MAST) score accurately identifies patients with NASH and significant fibrosis. J Hepatol. 2022;76(4):781–7. 10.1016/j.jhep.2021.11.012. 34798176

[27] Taru MG, Tefas C, Neamti L, et al. FAST and Agile—the MASLD drift: validation of Agile 3+, Agile 4 and FAST scores in 246 biopsy-proven NAFLD patients meeting MASLD criteria. PLoS One. 2024;19(5):e0303971. 10.1371/journal.pone.0303971. PMC11115280 38781158

[28] Lassailly G, Caiazzo R, Ntandja-Wandji LC, et al. Bariatric surgery provides long-term resolution of nonalcoholic steatohepatitis and regression of fibrosis. Gastroenterology. 2020;159(4):1290-1301.e5. 10.1053/j.gastro.2020.06.006. 32553765

[29] Noria SF, Shelby RD, Atkins KD, Nguyen NT, Gadde KM. Weight regain after bariatric surgery: scope of the problem, causes, prevention, and treatment. Curr Diab Rep. 2023;23(3):31–42. 10.1007/s11892-023-01498-z. PMC9906605 36752995

